# Impact of Sampling Time Variability on Tacrolimus Dosage Regimen in Pediatric Primary Nephrotic Syndrome: Single-Center, Prospective, Observational Study

**DOI:** 10.3389/fphar.2021.726667

**Published:** 2022-01-07

**Authors:** Lingfei Huang, Junyan Wang, Jufei Yang, Huifen Zhang, Yan Hu, Jing Miao, Jianhua Mao, Luo Fang

**Affiliations:** ^1^ Department of Pharmacy, The Children's Hospital, Zhejiang University School of Medicine, National Clinical Research Center for Child Health, Hangzhou, China; ^2^ Department of Nephrology, The Children's Hospital, Zhejiang University School of Medicine, National Clinical Research Center for Child Health, Hangzhou, China; ^3^ Department of Pharmacy, Cancer Hospital of the University of Chinese Academy of Sciences (Zhejiang Cancer Hospital), Institute of Basic Medicine and Cancer (IBMC), Chinese Academy of Sciences, Hangzhou, China

**Keywords:** sampling time, therapeutic drug monitoring, tacrolimus, nephrotic syndrome, pediatrics

## Abstract

**Background:** Tacrolimus (TAC) is an important immunosuppressant for children with primary nephrotic syndrome (PNS). The relationship between sampling time variability in TAC therapeutic drug monitoring and dosage regimen in such children is unknown.

**Methods:** In this single-center, prospective, observational study, we evaluated the sampling time variability, concentration error (CE), relative CE (RCE), and the impact of the sampling time on TAC dosage regimens in 112 PNS children with 188 blood samples. Nominal concentration (C_nom_) at 12-h after last TAC dose was simulated based on observed concentration (C_obs_) via previously published pharmacokinetic models, then CE and RCE were calculated. Inappropriate dosing adjustments resulting from deviated sampling time were evaluated based on a target C_nom_ of 5–10 ng/ml.

**Results:** We found that 32 and 68% of samples were respectively collected early (2–180 min) and delayed (4–315 min). Furthermore, 24, 22, 22, and 32% of blood samples were drawn within deviations of ≤0.5, 0.5–1, 1–2, and >2 h, respectively, and 0.3 ng/ml of CE and 6% RCE per hour of deviation occurred. Within a deviation of >2 h, 25% of C_obs_ might result in inappropriate dosing adjustments. Early and delayed sampling might result in inappropriate dose holding or unnecessary dose increments, respectively, in patients with C_obs_ ∼ 5 ng/ml.

**Conclusions:** Variable sampling time might lead to inappropriate dosing adjustment in a minority of children with PNS, particularly those with TAC C_obs_ ∼ 5 ng/ml collected with a deviation of >2 h.

## Introduction

Tacrolimus (TAC) is a cornerstone of immunosuppression after organ transplantation (Kidney Disease Improving Global Outcomes KDIGO Transplant Work Group, 2009;Ericson et al., 2017). It is now administered to children with primary nephrotic syndrome (PNS), a renal disease classically characterized by massive proteinuria, hypoalbuminemia, edema, and hypercholesterolemia (Lombel et al., 2013; Kaku et al., 2015; Subspecialty Group of Nephrology Society of Pediatrics Chinese Medical Association, 2017; Trautmann et al., 2020). Owing to the substantial individual variability of its pharmacokinetic profile, TAC doses are usually adjusted based on trough concentrations (C_0_) determined by therapeutic drug monitoring (TDM) (Brunet et al., 2019). Up to now, the consensus target C_0_ of TAC in pediatric PNS is 5–10 ng/ml during the first few months of treatment (usually first 6 months), recommended by several professional societies, including the organization of Kidney Disease: Improving Global Outcomes, and Chinese Medical Association (Lombel et al., 2013 ; Subspecialty Group of Nephrology Society of Pediatrics Chinese Medical Association, 2017 ).

An accurate C_0_ is crucial for precise TAC dosing; however, it is greatly dependent on appropriate sampling. Ideally, TAC should be administered twice daily at 12-h intervals. Thus, TAC dose is commonly adjusted to maintain the 12-h trough levels in the target range. However, in real clinical practice, the observed C_0_ (Cobs) was always not sampled exactly at 12-h after TAC administrating. For example, in posttransplant patients, sampling time deviation (STD) varies in the clinical setting from to 8–14 h, or even 2–25 h after the last dose ( Karaalp et al., 2004; Dasari et al., 2016; Strohbehn et al., 2018 ). Furthermore, the potential bias of clinical decisions caused by inaccurate C_0_ is considered financially inefficient and harmful (Wong et al., 2016; Strohbehn et al., 2018). Therefore, in pediatric PNS, TAC dose should be tailored based on the nominal C_0_ (C_nom_) of 12-h target C_0_ of 5–10 ng/ml.

Little is known about the variability of TAC STD in children with PNS. We previously simulated a STD-related concentration error (CE) and an inappropriate decision of dose tailoring *in silico* (Wang et al., 2020). An approximate 7% relative CE (RCE) per hour of STD occurred. Compared to delayed sampling, early sampling was associated with a larger RCE and higher risk of inappropriate dosing. However, STD induced inappropriate clinical dose decision is necessary to be clarified in real-world practice.

Meanwhile, due to the retrospective nature of the previously published study (Wang et al., 2020), some factors partly decided blood level of TAC, such as genotype of cytochrome P-450 system (CYP3A) family (Huang et al., 2020) and co-therapy medications of cost-saving agents (CYP3A inhibitors such as fluconazole, diltiazem, and Schisandra extract) prolonged TAC elimination (Zhao et al., 2016; Vanhove et al., 2017; Huang et al., 2019; Wang et al., 2021), were not involved. Furthermore, age (ontogeny) sometimes dominates the enzymatic functions (phenotype) in pediatric patients (Sun, et al., 2018), and the age-related pattern in CYP3A expression and activity is worth exploring.

Therefore, this single-center, prospective, observational study aimed to evaluated the variability of TAC STD, STD-related CE, and its potential adverse impact on clinical decisions regarding TAC dosage regimens involved factors of age, *CYP3A5* genotype, and CYP3A inhibitors.

## Methods and Design

### Patients and Ethics

This prospective observational study was conducted between April 2019 and March 2020 at a tertiary children’s teaching hospital in Hangzhou, Zhejiang Province, China.

We included consecutive patients who were diagnosed with PNS, aged <18 years, and then treated with a constant TAC dose for ≥3 days twice daily. Patients were excluded if they were unwilling to participate in the study, co-treated with traditional Chinese medicine except Wuzhi capsules [extract of *Schisandra sphenanthera*, which increased the TAC concentration (Chen et al., 2020)], or had serious infections or multiple organ injuries.

The Ethics Committee of the Children’s Hospital, Zhejiang University School of Medicine, approved the study (2019-IRB-039), which was registered in the Chinese Clinical Trial Registry (ChiCTR1900022724). All parents provided written informed consent before participation. All patient information was rendered anonymously before analysis.

### TDM, Genotyping, and Data Collection

Whole blood (2 ml) anticoagulated with EDTA was sampled for TDM, and the sample time was extracted from electronic medical records (EMR). Concentrations of TAC were determined using ARCHITECT i1000 chemiluminescent microparticle immunoassay kits (Abbott Laboratories, Chicago, IL, United States) as described by the manufacturer. The detection range was 2.0–30.0 ng/ml, and the inter- and intra-coefficients of variation were ≤10%. Total DNA was extracted, and the CYP3A5*3 genotype was assessed using the polymerase chain reaction as we previously described (Huang et al., 2020).

Data about TAC dosages, frequency, and intake times were analyzed. Inpatient information was extracted from the EMR. Data were collected from outpatients using social networking applications; a special two-dimensional code (QR code) was automatically sent to every patient, and related complete information were acquired by investigators. Information including therapeutic regimens, concomitant drugs (CYP3A4/5 inducers/inhibitors and Wuzhi capsules), and subsequent dose regimens by clinicians after TDM was also collected.

The interval between blood sampling and the last TAC dose was calculated. Early and delayed sampling was defined as blood drawn before and after the 12-h dosing intervals, respectively.

### Simulation of nominal TAC C_0_ and evaluation of concentration error of all measurable samples (Analysis set 1)

In this process, observed C_0_ (C_obs_) ≤ 2.0 ng/ml (the lower limit of quantitation) by TDM was excluded for its unmeasurable value. Then nominal C_0_ (C_nom_) at 12 h after the last TAC dose was simulated based on Cobs. A nonlinear mixed-effects model program, NONMEM®, version 7.4 (Icon, Inc., Lake Winola, PA, United States) and published population pharmacokinetic models (Huang et al., 2020; Chen et al., 2020) of Chinese children with PNS were applied for simulation (Supplementary Table S1).

The difference between the C_obs_ and C_nom_ was defined as the CE and calculated according to Eq. 1. The RCE was calculated using Eq. 2.

$$CE\left(ng/mL\right)=C_{obs}-C_{nom}$$ (1)

$$RCE\left(\%\right)=\frac{C_{obs}-C_{nom}}{C_{nom}}\times 100$$ (2)

### Evaluation of TAC dosing decision of subjects within the first 6 months of TAC treatment (Analysis set 2)

Because the consensus target C_0_ (5–10 ng/ml) only established for the first 6 months of TAC treatment in pediatric PNS (Lombel et al., 2013), the impact of STD on clinical dosing regimens was conducted among patients within 6 months after TAC treatment in this process.

We identified the following scenarios of inappropriate dosing decisions associated with STD: 1) inappropriate dose adjusting: when C_nom_ was within the target range (5–10 ng/ml) but C_obs_ was outside the target range and clinicians altered the TAC treatment; 2) inappropriate dose holding: when C_nom_ was outside the target range (<5 or >10 ng/ml) and C_obs_ was within it, but dosing was not altered. Inappropriate decisions were recorded, and subsequent therapy regimens and PNS management were followed up. The management of PNS was estimated as relapse and complete, partial, and or no remission.

### Statistical Analysis

Data were statistically analyzed using SPSS version 24.0 (IBM Corp, Armonk, NY, United States) and GraphPad Prism version 8.0 (GraphPad Software Inc., San Diego, CA, United States). Continuous data are presented as mean value ± standard deviation (SD) and median value with interquartile range (IQR) for normally and non-normally distributed variables, and analyzed using *t*-test and Mann-Whitney U test, respectively. Categorical data are presented as frequencies and proportions and were analyzed using chi-square tests. Correlations between STD and CE or RCE in total patients or subgroups of 0–3, 3–6, 6–9, 9–12, and 12–18 years old were investigated using linear regression. Differences were considered statistically significant at *p* < 0.05.

## Results

**FIGURE 1:**
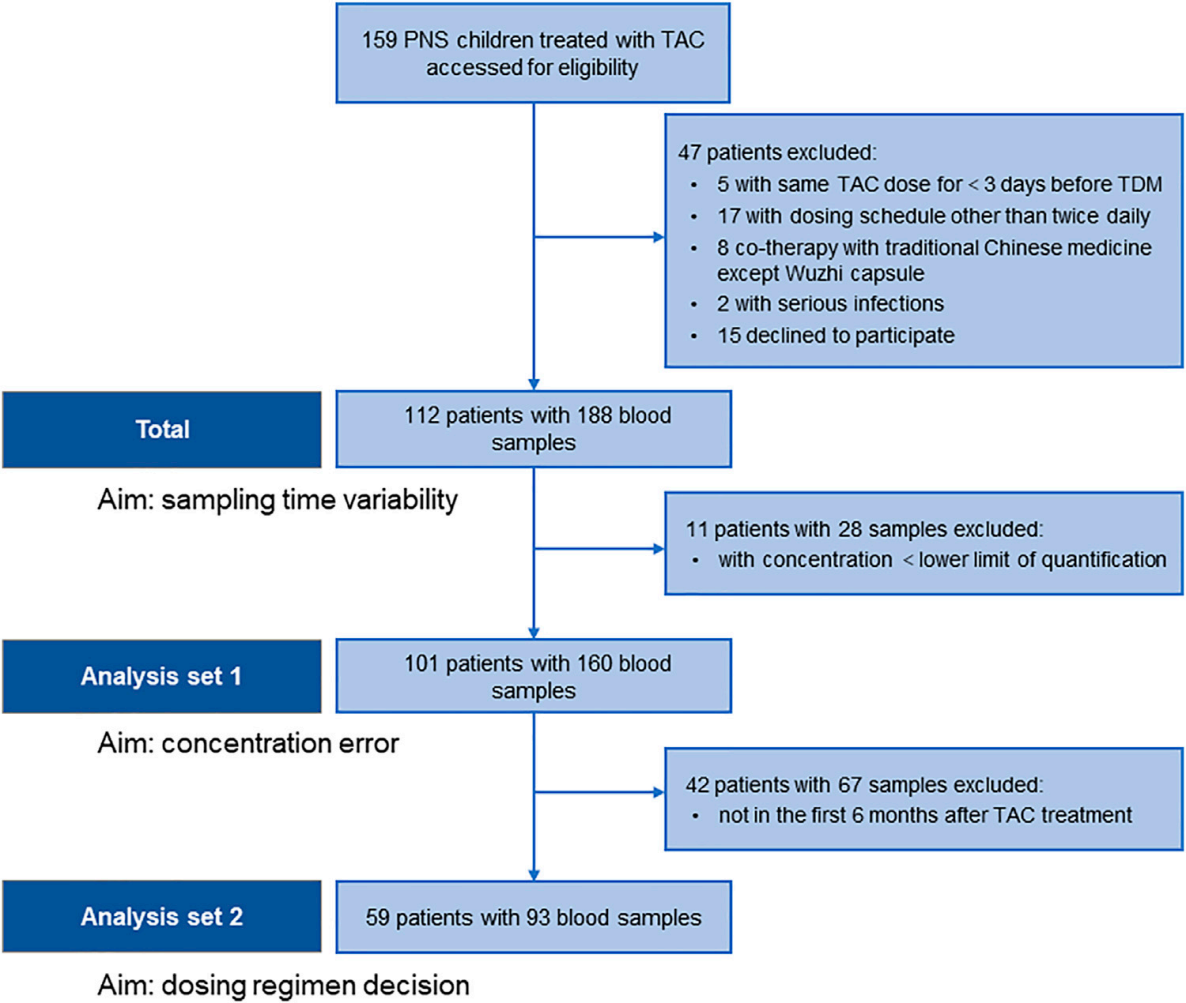
Flow chart of patient enrollment and data analyses.

We collected 188 blood samples from 112 patients with PNS, and then STD were estimated. Furthermore, CE and inappropriate clinical decision were estimated based on 160 measurable blood samples from 101 patients, and 93 samples of 59 patients within 6 months after initial treatment, respectively (Figure 1; Table 1).

**TABLE 1:** Clinical characteristics of patients.

Characteristics	Total	Analysis set 1^a^	Analysis set 2^a^
Patients (n)	112	101	59
Boys (n, %)	72 (64.3)	66 (65.3)	38 (64.4)
Age, years	8.2 ± 4.7 (1.3–17.0)	8.3 ± 4.6 (1.3–17.0)	8.1 ± 4.3 (1.5–16.4)
Weight, kg	32.5 ± 17.3 (9.0–75.8)	31.5 ± 16.0 (9.0–75.0)	29.5 ± 16.8 (9.0–67.0)
Albumin, g/L	28.8 ± 11.0 (9.7–49.8)	27.1 ± 10.9 (12.3–49.8)	27.6 ± 12.3 (12.3–49.8)
Serum creatinine, µmol/L	55.2 ± 15.3 (30.0–122.0)	54.8 ± 15.4 (30.0–122.0)	54.5 ± 16.4 (30.4–122.0)
CYP3A5*3*3 (n, %)	68 (61.0)	94 (58.8)	60 (64.5)
Blood samples (n)	188	160	93
Tacrolimus daily dose, mg	1.6 ± 0.7 (0.5–4.0)	1.7 ± 0.7 (0.5–4.0)	1.6 ± 0.6 (0.5–3.0)
Tacrolimus daily dose, µg/kg	61.7 ± 23.8 (11.1–142.8)	63.1 ± 23.6 (11.1–142.8)	66.1 ± 23.0 (16.9–142.8)
Observed concentration, ng/mL	5.5 ± 3.0 (2.0–21.4)	5.5 ± 3.0 (2.0–21.4)	5.6 ± 3.1 (2.0–21.4)
Nominal concentration, ng/mL	—	5.8 ± 3.0 (1.9–21.4)	5.9 ± 3.1 (1.9–21.4)
Early sampled (n, %)	60 (31.9)	50 (31.2)	32 (34.0)
Co-therapy medications (n, %)			
Azole antifungal agents^b^	2 (1.1)	1 (0.6)	0 (0.0)
Diltiazem^b^	30 (16.0)	20 (12.5)	7 (7.4)
Wuzhi capsule^b^	94 (50.0)	90 (56.3)	48 (51.6)

^a^Analysis set 1, samples with measured TAC, for concentration error evaluation; Analysis set 2, samples within 6 months after initial treatment for inappropriate dosing decisions evaluation.

b Number of samples (percentage).

### Sampling Time Deviations of TAC TDM

**FIGURE 2:**
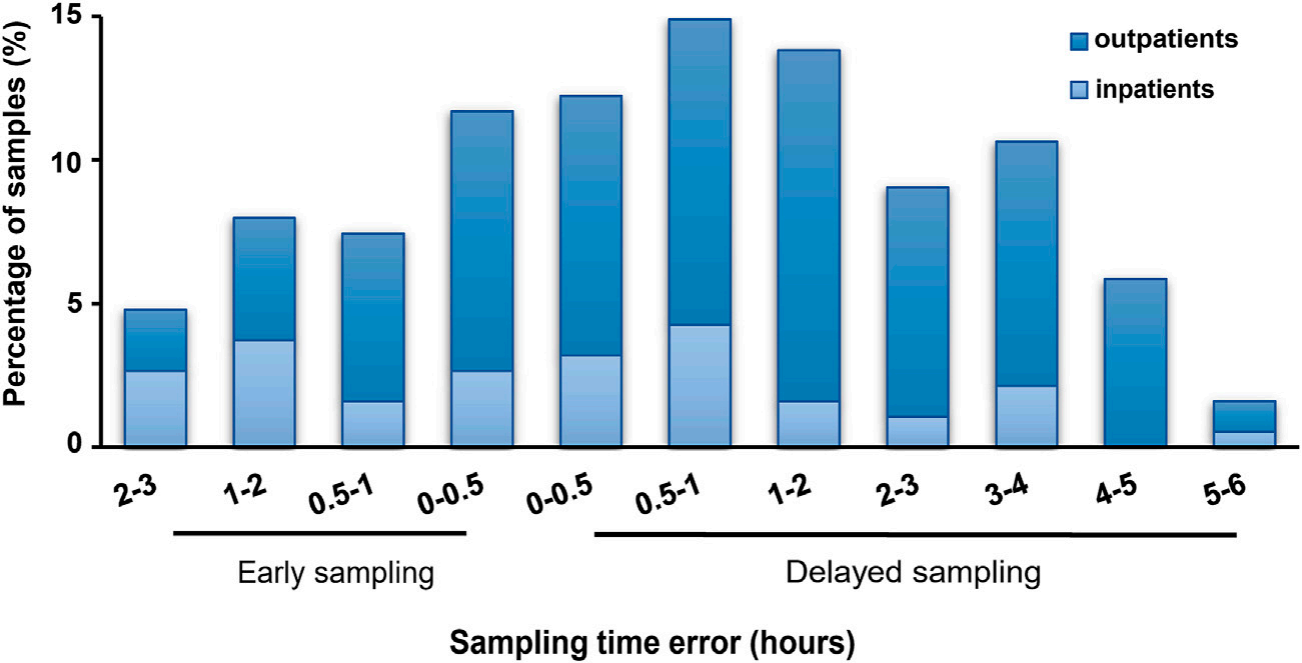
Sampling time deviations of tacrolimus therapeutic drug monitoring in pediatric primary nephrotic syndrome. All samples (*n* = 188); dark blue: samples from outpatients (*n* = 144); light blue: samples from inpatients (*n* = 44).

Among all samples, 128 (68.1%) were delayed while 60 (31.9%) were early sampled, and 76.6% were collected from outpatients (dark blue in Figure 2). Approximately a quarter (23.9%) and a half (46.3%) samples were collected within STD ≤0.5 h and ≤1 h, respectively. Meanwhile, the STD was longer in delayed than in early samplings (median, 70; range, 4–315; IQR, 30–180 min vs median, 45; range, 2–180; IQR, 26–90 min; *p* < 0.05).

### Impact of Sampling Time on TAC Concentration Error

**FIGURE 3:**
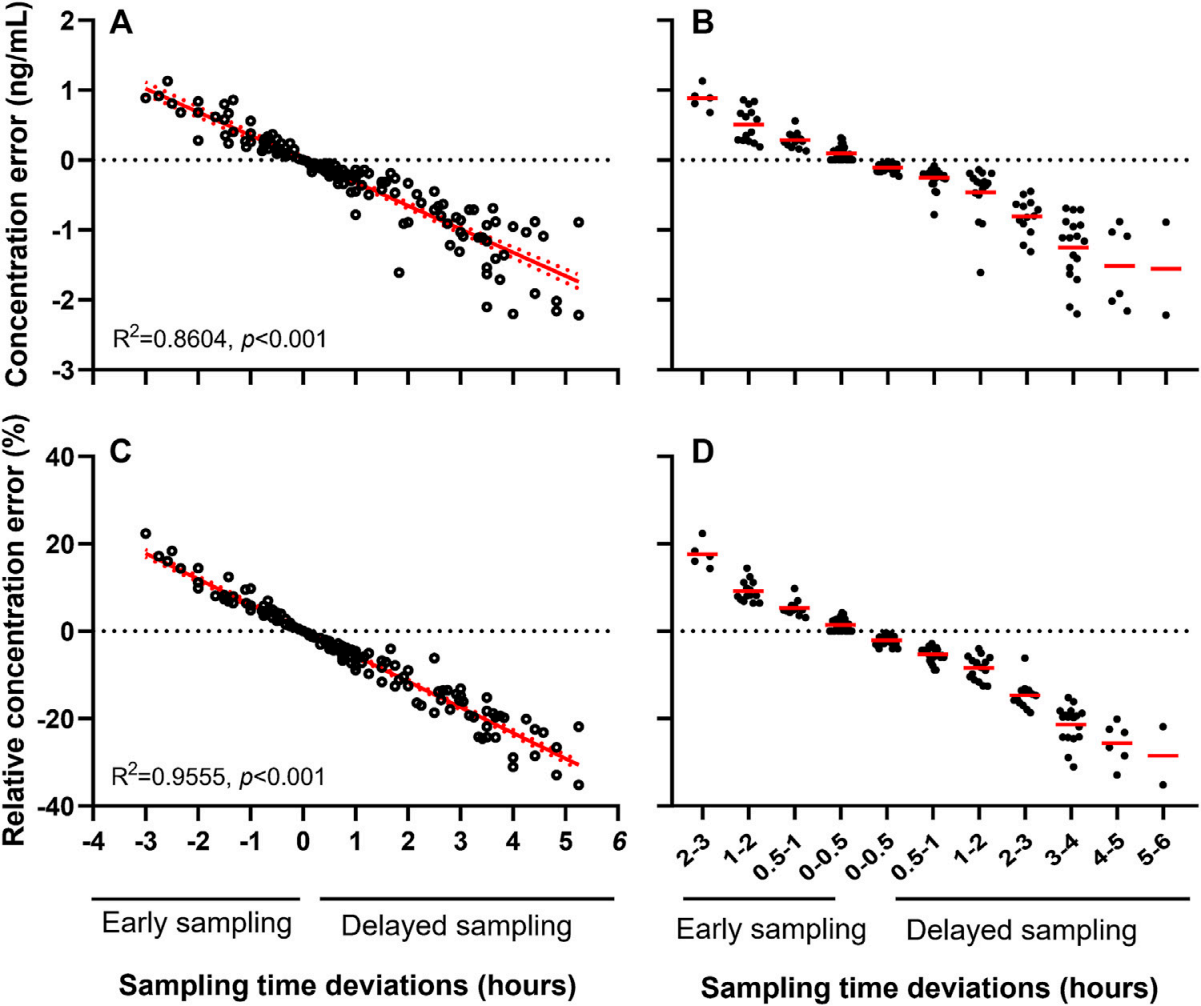
Sampling time deviation induced concentration error **(A, B)** and relative concentration error **(C, D)** of tacrolimus.

The 160 samples with measured C_obs_ were used for C_nom_ simulation via NONMEM. Mean C_obs_ and C_nom_ were 5.5 ± 3.0 and 5.8 ± 3.0 ng/ml, respectively (Table 1, “Analysis set 1”). The CE and RCE ranges in early samples were 0–1.1 ng/ml (Figure 3A) and 0.2–22.4% (Figure 3C), respectively, and in delayed samples were 0–2.2 ng/ml (Figure 3A), and 0.3–32.9% (Figure 3C), respectively. Linear relationships were strong between STD and CE as well as RCE (correlation coefficients for CE and RCE, 0.860 and 0.956, respectively; *p* < 0.001 for both). Approximately, 0.3 ng/ml of CE (Figure 3B) and 5.9% of RCE (Figure 3D) occurred for every hour of STD.

Meanwhile, considering the potential influence of different age on the pharmacokinetics of TAC, correlation of RCE and STD was further analyzed in various subpopulation with 0–3, 3–6, 6–9, 9–12, and 12–18 years old. RCE by 1-h STD was increased from 5.35% of 0–3 aged patients to 6.16% of 12–18 aged patients (Supplementary Figure S1A–E). Moreover, it was disclosed that RCE per hour of STD was significantly linked to age (*p* = 0.0134, r = 0.950) (Supplementary Figure S1F).

### Impact of Sampling Time on TAC Dosing Decision

TAC dosing decision after TDM in 93 samples collected within 6 months after initial treatment (Table 1, “Analysis set 2”) were evaluated in 59 patients. Among 93 samples, 9 paired C_obs_ and C_nom_ failed to consistently within target range, and occurred at scenarios with STD of 1–2 h (2 samples), 2–3 h (4 samples), 3–4 h (2 samples), and 4–5 h (1 sample). Furthermore, 6 of the 9 TAC dose decisions were inappropriate ( Table 2 ). The respective CE and RCE ranges of −2.2–1.0 ng/ml and −33.3–25.0% in these incidents indicated a high incidence of inevitable inaccurate clinical decisions based on TAC dose adjustments.

**TABLE 2:** Inappropriate tacrolimus dose regimen decisions caused by sampling time deviations.

Patient^a^	TAC DD (mg)	Weight (kg)	STD (hours)	Trough concentration (ng/ml)			Clinical dose regimen decision		Events after TDM
				Observed	Nominal	RCE	Observed	Nominal	
Early sampled (over-estimated concentration)									
No. 1	1.0	12.5	3.0	5.0	4.0	25.0%	Holding	Increased	Relapse after 15 days, then combined with WZ
No. 2	1.0	17.4	2.5	5.2	4.4	18.2%	Holding	Increased	Partial remission, then combined with WZ
No. 3	2.0	36.7	2.3	5.4	4.7	14.9%	Holding	Increased	Partial remission, then combined with RTX
Delayed sample (under-estimated concentration)									
No. 4	0.5	11.5	2.9	4.9	5.6	−12.5%	Increased	Holding	Complete remission
No. 5	1.5	21.0	3.7	4.4	5.8	−24.1%	Increased	Holding	Complete remission
No. 6	2.0	54.0	4.8	4.4	6.6	−33.3%	Increased	Holding	Complete remission

TAC, tacrolimus; DD, daily dose; STD, sampling time deviation; RCE, relative concentration error; TDM, therapeutic drug monitoring; WZ, Wuzhi capsule; RTX, rituximab.

a All patients were CYP3A5*3/*3 carriers (CYP3A5 nonexpressers, slow metabolizer) except patient No.4 (CYP3A5*1/*3 carrier); and there were no co-therapy drugs when TDM, sampled except patient No.3 (combined with Wuzhi capsule).

Two scenarios, inappropriate TAC dose holdings for early sampling and unnecessary dose increases for delayed sampling, were observed. Doses were inappropriately withheld in three children (Table 2, Nos. 1–3) with overestimated C_obs_, and this led to unsatisfactory clinical efficacy. PNS relapsed in one child at 15 days after TDM, and the other two children were partial remission, and the regimens were subsequently modified by combination with Wuzhi capsules or rituximab. Three children with inappropriately increased instead of being maintained with under-estimated C_obs_, although the clinical outcomes were as expected (Table 2, Nos. 4–6).

Notably, all these samples had C_obs_ ∼5 ng/ml and were collected with STD >2 h; that is, the probability of inappropriate incidents was much higher when samples were collected with STD >2 h (25.0%, 6 in 24 samples) than ≤2 h (0 in 69 samples). As this study included only Chinese children, among whom, only one was a CYP3A5*1/*3 carrier; therefore, the influence of genetic polymorphisms was not statistically comparable. Similarly, only one child received Wuzhi capsules, and the effect of co-administered drugs was not estimated.

## Discussion

TAC dosage regimens should be optimized based on TDM. However, obtaining accurate TAC concentrations in clinical practice is challenging owing to unsatisfactory patient compliance, measurement uncertainty, circadian rhythms, and other factors (Ertas et al., 2002;Eaton et al., 2018; Fontova et al., 2021). An important and prevalent cause of measurement uncertainty is STD (Saint-Marcoux et al., 2003; Choi et al., 2013; Moeller et al., 2014;Jakobsen et al., 2017). The present study is the first to determine the prevalence of TAC sampling time variability in pediatric patients with PNS and to elucidate possible STD-related concentration errors and inappropriate dose adjustments.

As disclosed in the present study, about half and one third of blood samples, respectively, were collected with STD of > 1 h and > 2 h. STD was common in the TDM of TAC. It was similar to the findings of adult organ transplantation ( Karaalp et al., 2004; Dasari et al., 2016;Strohbehn et al., 2018 ). However, a specific time frame for collecting blood samples of TAC C_0_, has not been defined owing to the lack of evidence regarding a relationship between STD, and adverse events. Generally, a “clinically ideal” state that allows for a tolerance of 1 h around each TDM event has gained experiential acceptance (Karaalp et al., 2004). Notably, in children with PNS, a slight bias (CE 0.3 ng/ml and RCE 6%) within this interval did not result in subsequent inappropriate dose adjustment. Therefore, this “ideal” time frame of ±1 h might be clinically applicable.

Inappropriate decisions on TAC dose adjustment induced by STD within deviations of ≤2 h were not generally remarkable. However, clinicians should be aware of patients with C_obs_ around the lower limit of the C_0_ target, particularly those with C_obs_ ∼ 5 ng/ml, and sampled within STD >2 h, for the excessive risk of inappropriate dose-holding induced unsatisfactory TAC effect in early sampled patients, and inappropriate dose-increase induced unnecessary costs in delayed sampled patients, respectively. Therefore, a repeat TDM at an accurate sampling time might be necessary. Moreover, educating patients and their family caregivers about sampling adherence is crucial (Shi et al., 2020). Medical workers also require education about pharmacokinetics and TDM, and laboratory technicians and nurses should be reminded about correct sampling times and their importance (Schiff et al., 2003;Chen et al., 2003).

To preliminarily disclosed the cause of individual variate of CE, the role of *CYP3A5*, the most efficient metabolizing enzyme of TAC, and was evaluated. CYP3A5*3 genetic polymorphism is widely regarded as an essential factor to the high individual variability of TAC (Woillard et al., 2017; Brunet et al., 2019; Mendrinou et al., 2020; van Gelder et al., 2020), and pediatric PNS patients with the CYP3A5*1 allele (rapid metabolizer) have lower TAC exposure than those who do not express *CYP3A5* (CYP3A5*3/*3, slow metabolizer) (Huang et al., 2019). Meanwhile, the metabolism of TAC is inhibited by CYP inhibitors. However, the hypothesized greater effect of STD on a TAC rapid metabolizer without a combination of CYP inhibitors was not observed, and the long-term outcomes did not seem remarkably impaired. It might partly be attributed to most Chinese are slow metabolizers, and the negative influence of STD might be moderate in Chinese pediatric patients with PNS, despite limited data.

Besides CYP3A5, other metabolic CYP enzymes of TAC, such as CYP3A4 and CYP3A7 (Barbarino et al., 2013), can be involved in the further study to fully disclose the individual genetic variate of STD caused CE. Moreover, we found increased RCE along with aging in pediatric PNS patients. It was consistent with our previous finding *in silico* (Wang et al., 2020). This phenomenon may be particularly due to the age-related pattern in CYP3A protein expression and activity, and age (ontogeny) sometimes dominate the enzymatic functions (phenotype) in pediatric patients (Sun, et al., 2018). Activity of CYP3A4 is very low before birth but increases rapidly, whereas CYP3A7 displays a significant decline in postnatal periods. For CYP3A5, expression and function stay stable from gestation through adulthood. However, the ontogeny was not enough to explain it, for other factors as high body weight, daily dose, and corticosteroid dose, etc. may also related to increased RCE (Wang et al., 2020). But, nevertheless, the potential influence of different age on RCE should be valued.

This study has some limitations. First, we simulated C_nom_ based on previous population pharmacokinetic models, and bias towards real C_nom_ should be considered. Second, the impact of STD on TAC toxicity and adverse events was not investigated because of having few samples with TAC concentrations >10 ng/ml. Third, most C_obs_ below limit of quantitation were in delayed samples and were excluded during C_nom_ simulation because of the lack of exact values, which might weaken their clinical implications. Fourth, we included only patients within 6 months after TAC treatment who reached the target C_0_ of 5–10 ng/ml, because a specific C_0_ target for PNS after 6 months has not been recommended. Moreover, whether inaccurate TAC values could affect the long-term outcomes of pediatric PNS remains undefined, nonetheless, we linked them to the closely followed dosage decision; thus, these mysteries need to be resolved in studies with multi-center and large-sample clinical studies.

## Conclusions

The present study found that the time of collecting blood samples from children with PNS to measure TAC varied in the clinical setting, and could result in a TAC concentration error as high as 30%. Although the impact of inappropriate sampling times on clinical decisions might have been moderate, STD >2 h associated with inaccurate C_0_ exposure should be worthy of attention.

## Data Availability

The original contributions presented in the study are included in the article/Supplementary Material, further inquiries can be directed to the corresponding authors.
