# Corrigendum: Impact of sampling time variability on tacrolimus dosage regimen in pediatric primary nephrotic syndrome: Single-center, prospective, observational study

**DOI:** 10.3389/fphar.2022.1051794

**Published:** 2022-10-14

**Authors:** Lingfei Huang, Junyan Wang, Jufei Yang, Huifen Zhang, Yan Hu, Jing Miao, Jianhua Mao, Luo Fang

**Affiliations:** ^1^ Department of Pharmacy, The Children’s Hospital, Zhejiang University School of Medicine, National Clinical Research Center for Child Health, Hangzhou, China; ^2^ Department of Nephrology, The Children’s Hospital, Zhejiang University School of Medicine, National Clinical Research Center for Child Health, Hangzhou, China; ^3^ Department of Pharmacy, Cancer Hospital of the University of Chinese Academy of Sciences (Zhejiang Cancer Hospital), Institute of Basic Medicine and Cancer (IBMC), Chinese Academy of Sciences, Hangzhou, China

**Keywords:** sampling time, therapeutic drug monitoring, tacrolimus, nephrotic syndrome, pediatrics

In the published article, there was an error in Affiliations 1, 2, 3.

In affiliation 1, instead of “Department of Pharmacy, The Children’s Hospital, National Clinical Research Center for Child Health, Zhejiang University School of Medicine, Hangzhou, China”, it should be “Department of Pharmacy, The Children’s Hospital, Zhejiang University School of Medicine, National Clinical Research Center for Child Health, Hangzhou, China”.

In affiliation 2, instead of “Department of Nephrology, The Children’s Hospital, National Clinical Research Center for Child Health, Zhejiang University School of Medicine, Hangzhou, China”, it should be “Department of Nephrology, The Children’s Hospital, Zhejiang University School of Medicine, National Clinical Research Center for Child Health, Hangzhou, China”.

In affiliation 3, instead of “Department of Pharmacy, Institute of Basic Medicine and Cancer (IBMC), Chinese Academy of Sciences, Cancer Hospital of the University of Chinese Academy of Sciences (Zhejiang Cancer Hospital), Hangzhou, China”, it should be “Department of Pharmacy, Cancer Hospital of the University of Chinese Academy of Sciences (Zhejiang Cancer Hospital), Institute of Basic Medicine and Cancer (IBMC), Chinese Academy of Sciences, Hangzhou, China”.

The authors apologize for this error and state that this does not change the scientific conclusions of the article in any way. The original article has been updated.

