# Supplementary figures and images for: Impact of Sampling Time Variability on Tacrolimus Dosage Regimen in Pediatric Primary Nephrotic Syndrome: Single-Center, Prospective, Observational Study
Source: Front Pharmacol. 2022 Jan 7;12:726667. doi: 10.3389/fphar.2021.726667 (PMC8776711; doi:10.3389/fphar.2021.726667)

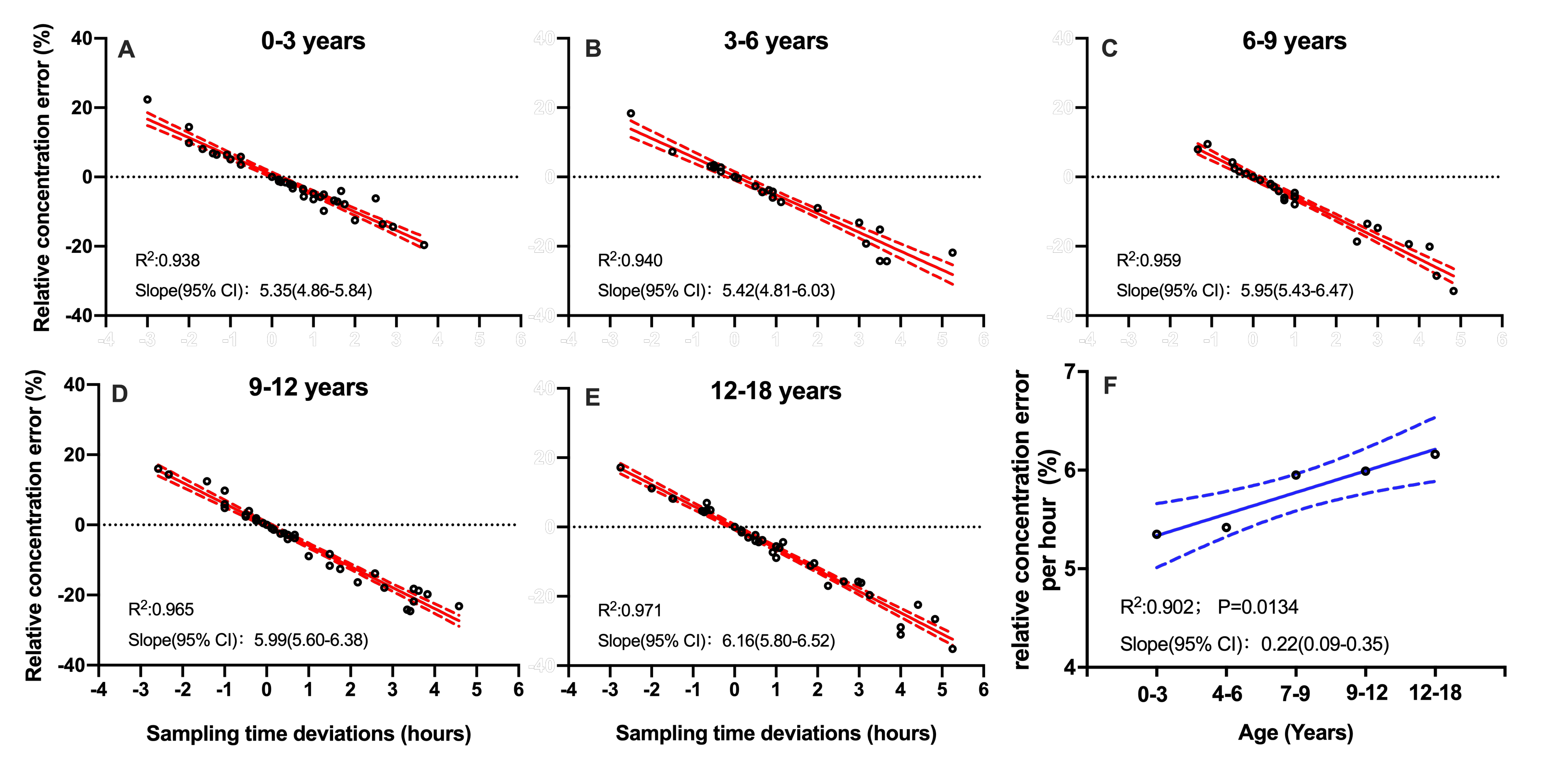

Supplement: Supplementary file 1 [file Image1.TIFF]
