# Supplementary material for: Impact of Sampling Time Variability on Tacrolimus Dosage Regimen in Pediatric Primary Nephrotic Syndrome: Single-Center, Prospective, Observational Study
Source: Front Pharmacol. 2022 Jan 7;12:726667. doi: 10.3389/fphar.2021.726667 (PMC8776711; doi:10.3389/fphar.2021.726667)
Supplement: Supplementary file 2 [file DataSheet1.docx]

**Supplementary**

Two TAC population pharmacokinetic models in Chinese children with PNS previously designed were applied for the simulation of the TAC concentrations. The models were built with one compartment model with first-order elimination and the formula of clearance (CL/F) in the final model was presented as Equations 1-2. One model was constructed by our team (Equation 1) ^[1]^, and another by Xiao Chen *et al*. (Equation 2) ^[2]^.

CL/F (L/h) = 7.13×(DD/1.5)^0.225^×(WT/25)^0.265^×1.394^CYP3A5^×0.538^Azole^×0.88^DTZ^ (1)


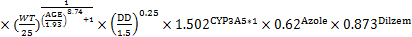


CL/F (L/h) = 20.1×(WT/70)^0.75^×1.5^CYP3A5^× (1-0.303×WZ) (2)

where WT is the body weight, DD is the daily dose of TAC. When patients carry *CYP3A5*3*3* genotype, the value of *CYP3A5* was 0, whereas *vice versa* was 0. When combined with azole antifungal agents, diltiazem or Wuzhi capsule, the value of Azole, DTZ or WZ was 1, whereas *vice versa* was 0.

The bioassays used in these two studies were enzyme multiplied immunoassay technique (EMIT), and in our study was chemiluminescent microparticle immunoassay (CMIA). There were systematic biases between the different methods, and the concentrations were converted to their corresponding equivalents according to Equation 3, which are from large clinical studies in adult renal transplant recipients:

CMIA = 0.93 × EMIT + 0.36 (3)

For each scenario, 1000 times of simulation was performed. Characteristics of model covariates for concentration simulations are listed in **Table S1**.

**References:**

1. Huang L, Liu Y, Jiao Z, et al. Population pharmacokinetic study of tacrolimus in pediatric patients with primary nephrotic syndrome: A comparison of linear and nonlinear Michaelis-Menten pharmacokinetic model. *Eur J Pharm Sci*. 2020; 143:105199.
2. Chen X, Wang DD, Xu H, et al. Optimization of initial dosing scheme of tacrolimus in pediatric refractory nephrotic syndrome patients based on CYP3A5 genotype and coadministration with wuzhi-capsule. *Xenobiotica*. 2020;50(5):606‐613.
3. Bazin C, Guinedor A, Barau C, et al. Evaluation of the architect tacrolimus assay in kidney, liver, and heart transplant recipients. J Pharm Biomed Anal 2010; 53: 997–1002.

**Table S1.** Characteristics of model covariates for concentration simulations

| **Covariates** |  |
| --- | --- |
| Blood samples (n) | 160 |
| Tacrolimus daily dose, mg | 1.7 ± 0.7 (0.5-4.0) |
| Body weight, kg | 31.5 ± 16.0 (9.0-75.0) |
| *CYP3A5*3*3* | 94 (58.8) |
| Co-therapy medications (n, %) |  |
| Azole antifungal agents | 1 (0.6) |
| Diltiazem | 20 (12.5) |
| Wuzhi capsule | 90 (56.3) |


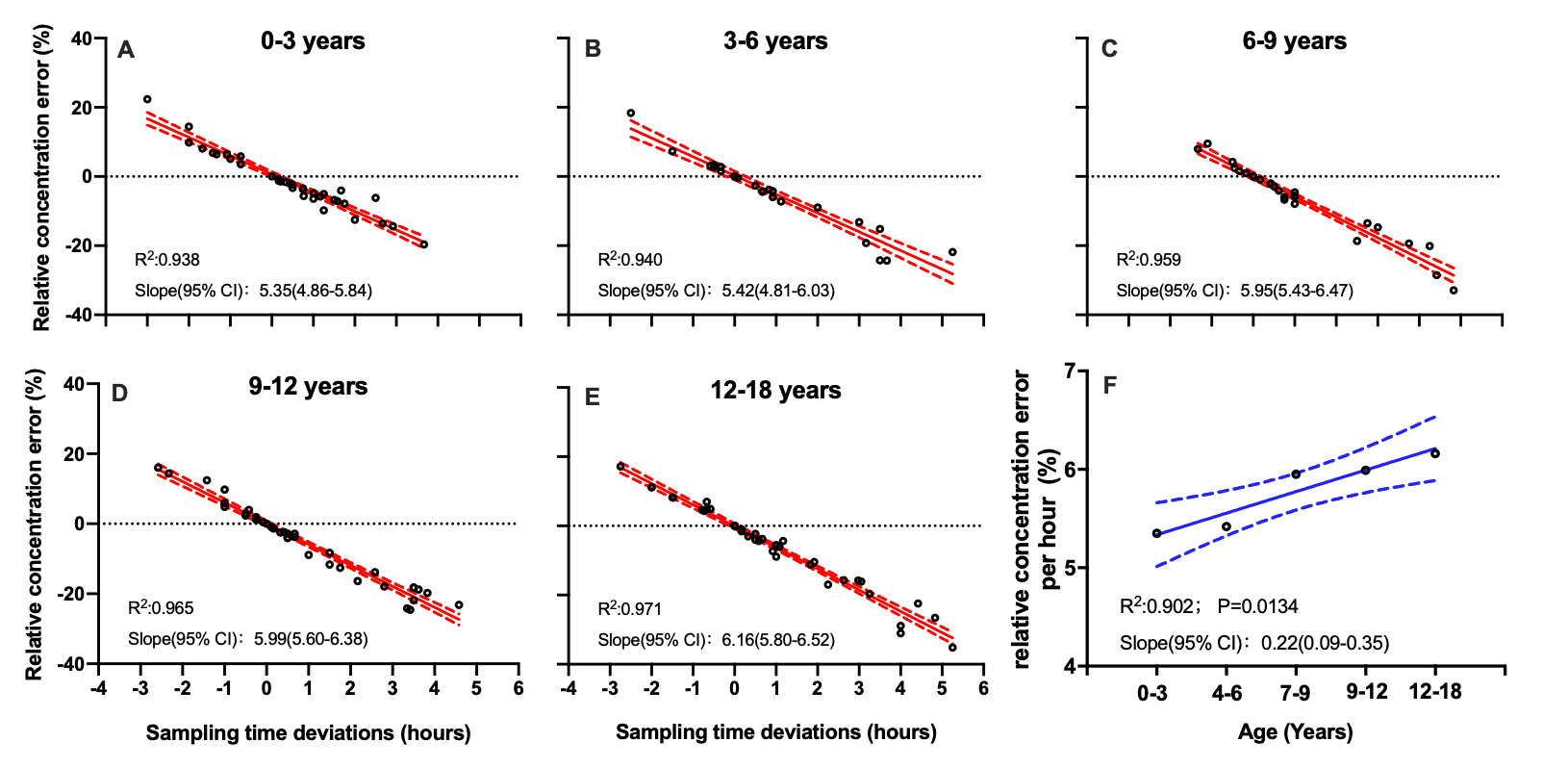


**Figure S1**. Correlation of relative concentration error (RCE) per hour of sampling time deviation (STD) based on age.

(A)-(E): increased RCE was related to aging; (F): RCE per hour of STD was significantly linked to age.
